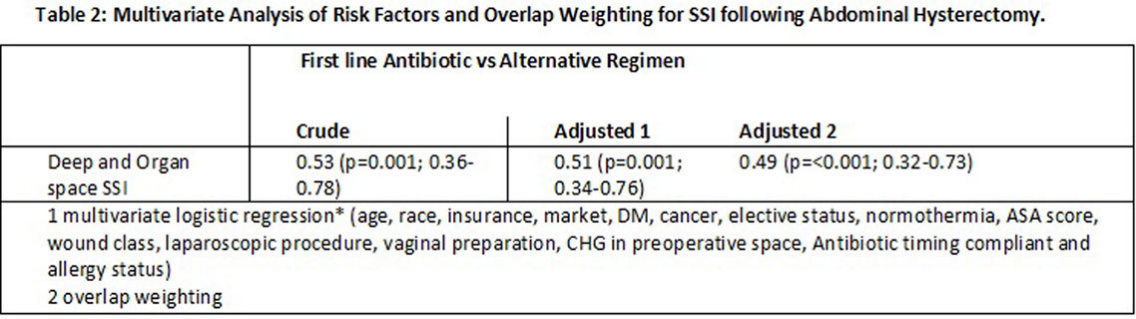# 114 Improving Empiric Antibiotic Selection for Patients with Cancer Hospitalized with Infection: Secondary Analysis of the INSPIRE Trials

**DOI:** 10.1017/ash.2026.10457

**Published:** 2026-06-23

**Authors:** Anupama Neelakanta, Tsai-Wei Wang, Brittany Lees, Werner Bischoff, Preston Miller, Catherine Passaretti

**Affiliations:** 1 Atrium Health; 2 Wake Forest University School of Medicine

## Abstract

**Introduction:** The American College of Obstetricians and Gynecologists (ACOG) and Society of Healthcare epidemiology (SHEA) recommend cephalosporins as the standard antibiotic for preoperative prophylaxis for abdominal hysterectomy (AH-includes Laparoscopic and open cases) . However, anaerobic organisms are prevalent in vaginal and intraabdominal flora and can contribute to development of surgical site infections (SSIs). Limited data suggest that including anaerobic coverage may reduce SSI risk. In May 2024, we implemented anaerobic containing regimens as first-line preoperative antibiotic prophylaxis for all AH cases. This study evaluates whether adding anaerobic coverage to the preoperative antibiotic protocol reduces National Healthcare Safety Network (NHSN)-defined SSI after AH surgeries. **Methods:** We conducted a retrospective cohort study of all patients who underwent inpatient or outpatient from January 2023 through September 2025. Preoperative antibiotic choice and timing as well as patient demographics and comorbidities were obtained from medical records. SSI were determined by NHSN definitions by trained infection preventionists. First line antibiotics with anaerobic coverage were defined as cefazolin plus metronidazole, or vancomycin plus aztreonam plus metronidazole in patients with penicillin allergies. SSI in patients who received first line antibiotics with anaerobic coverage were compared to those who received alternative antibiotic regimens (Around 80% of alternative regimen consisted of only cefazolin). Multivariate logistic regression and overlap weighting methods were applied to evaluate the independent impact of first-line therapy. **Results:** Among 11,444 hysterectomy procedures, the overall SSI rate was 1% (n=109 cases). Unadjusted SSI rate in patients receiving first-line antibiotics was significantly lower than those receiving alternate prophylaxis (0.7% vs 1.3 %, p =0.001). Individuals who received first-line antibiotics were generally younger, more frequently identified as Caucasian, and exhibited greater rates of diabetes and cancer compared to those who received alternative regimens. Patients who received first line antibiotics also had longer surgeries, a higher frequency of elective and laparoscopic procedures, and higher rates of adherence to preoperative protocols such as chlorhexidine bathing, vaginal preparation, and maintenance of normothermia (see Table 1). After adjusting for baseline differences between groups through multivariate logistic regression and overlap weighting, first-line therapy was associated with a 50% reduction in SSI risk compared to alternative regimens (OR 0.51; 95% CI 0.34–0.76; p=0.001). (Table 2) **Conclusion:** Compared to alternative regimens for preoperative antibiotic prophylaxis, the routine inclusion of metronidazole to cefazolin was associated with a reduced risk of SSI following AH surgeries even after adjusting for demographic, procedural and clinical differences between groups.

**Figure:**
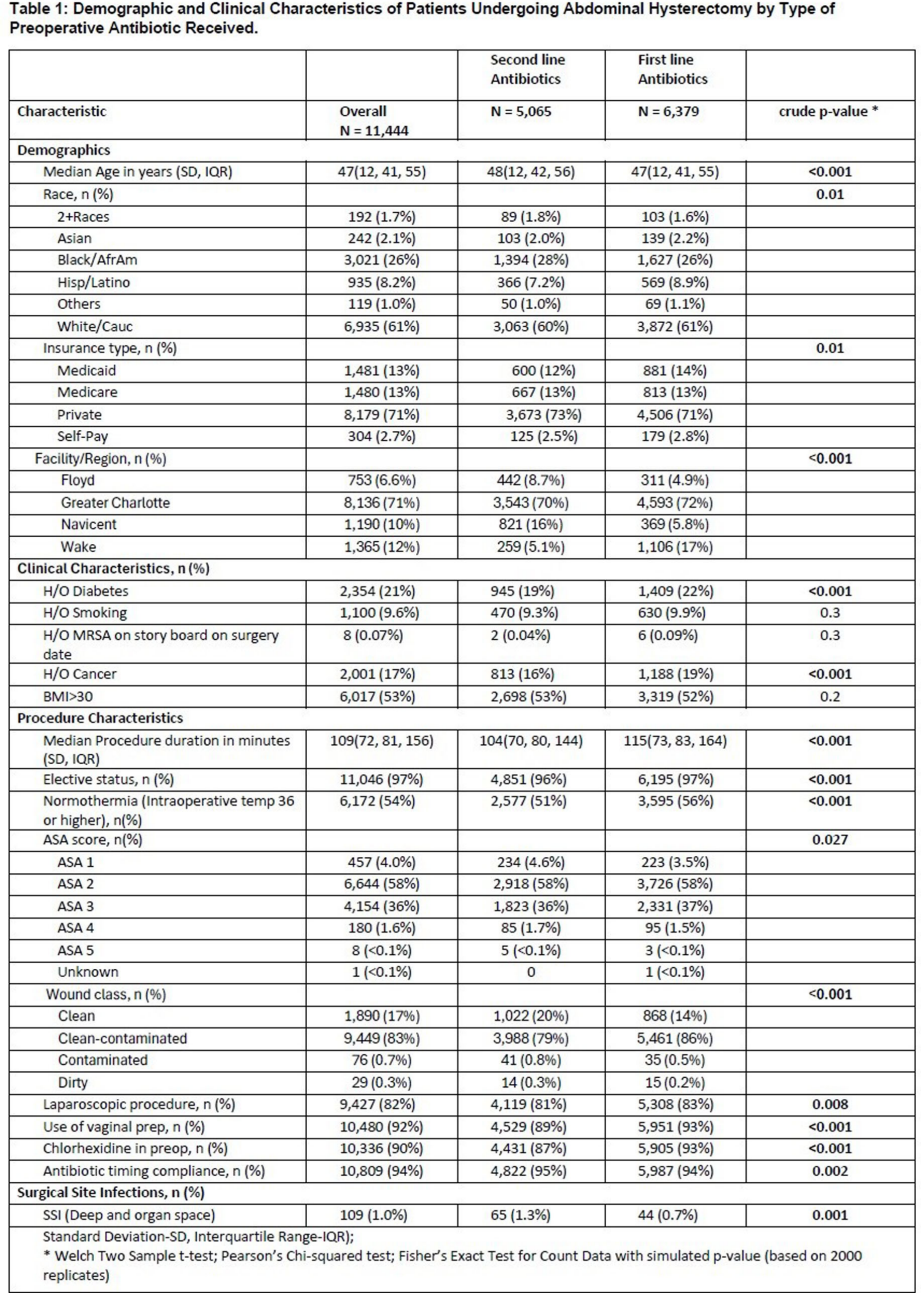

**Figure:**